# The socio-environmental production of malaria in three municipalities in the Carajás region, Pará, Brazil

**DOI:** 10.11606/s1518-8787.2021055003463

**Published:** 2021-10-18

**Authors:** Alba Lucia Ribeiro Raithy Pereira, Claudia do Socorro Carvalho Miranda, Juan Andrade Guedes, Rafael Aleixo Coelho de Oliveira, Pedro Silvestre da Silva Campos, Vera Regina Da Cunha Menezes Palácios, Camylle Maia Costa Faria, Tainara Carvalho G. M. Filgueiras, Roberto Carlos Figueiredo, Nelson Veiga Gonçalves

**Affiliations:** I Universidade do Estado do Pará Centro de Ciências Biológicas e da Saúde Departamento de Saúde Comunitária BelémPA Brasil Universidade do Estado do Pará. Centro de Ciências Biológicas e da Saúde. Departamento de Saúde Comunitária, Laboratório de Epidemiologia e Geoprocessamento da Amazônia. Belém, PA, Brasil; II Universidade Federal Rural da Amazônia Instituto Ciberespacial BelémPA Brasil Universidade Federal Rural da Amazônia. Instituto Ciberespacial. Belém, PA, Brasil

**Keywords:** Malaria, epidemiology, Land Use, Anthropological Factors, Risk Factors, Spatial Analysis

## Abstract

**OBJECTIVE:**

To analyze the environmental production of malaria in the municipalities of Marabá, Parauapebas, and Canaã dos Carajás, in Pará, from 2014 to 2018.

**METHODS:**

This ecological, cross-sectional study used epidemiological data in the *Sistema de Informações de Vigilância Epidemiológica da Malária* (Malaria Epidemiological Surveillance Information System) from the *Secretaria de Saúde do Estado do Pará* (State of Pará Health Department), cartographic data from the Brazilian Institute of Geography and Statistics (IBGE), and environmental data in the *Projeto TerraClass* (TerraClass Project) from the National Institute of Space Research (INPE). Statistical analyses used the chi-square test, while the spatial ones, the kernel and Moran’s (I) global bivariate techniques.

**RESULTS:**

We analyzed a total of 437 confirmed cases of malaria in the selected area and period. The highest percentage of cases occurred among male miners and farmers, living in rural areas; *Plasmodium vivax* was the most frequent species; and the most used diagnosis, the thick drop/smear. We also observed a heterogeneous distribution of the disease — with evidence of spatial dependence between incidence areas and different forms of land use, and spatial autocorrelations related to the high variability of anthropic activities in the municipalities.

**CONCLUSION:**

The environmental production of malaria relates mainly to cattle production and mining — anthropisms related to land use and occupation in the observed municipalities. Spatial data analysis technologies sufficed for the construction of the epidemiological scenario of the disease.

## INTRODUCTION

Malaria is an infectious, acute febrile disease of parasitic etiology, whose occurrence entails humans, protozoa of the *genus Plasmodium,* and infected female mosquitos of the genus *Anopheles* (Diptera: *Culicidae*). The disease is considered the main parasitic one in the world, with a significant number of cases in tropical and subtropical countries^1^.

According to the World Health Organization (WHO), malaria is endemic in 105 countries, with 228 million cases reported in 2018, causing 405,000 deaths. The Pan American Health Organization identified regions in Colombia, Ecuador, Venezuela, and Brazil in which the disease is expanding^4^.

Despite the decreasing number of cases in Brazil since the 1960s, malaria is still highly endemic, and considered a complex public health problem due to the country’s different environmental, socioeconomic and demographic characteristics — especially in the Amazon, which reported, in 2018 alone, more than 170,000 cases^2^.

The state of Pará, located in the eastern part of the Amazon, reported around 115,000 cases from 2014 to 2018; and further increase is expected. The growing numbers, especially in the municipalities of Canaã dos Carajás, Marabá, and Parauapebas, in the Carajás Integration Region, relate to environmental changes in the area, especially those linked to land use and cover. The impact of systematic, continuous environmental degradation makes these three municipalities emblematic of the environmental production of malaria^5^_._

Among its main causes is the unsustainable, socially unfair economic exploitation of natural resources, observed repeatedly in these municipalities; activities such as logging, farming, cattle production, mining and infrastructure have caused intense migratory flows, extensive deforestation and, consequently, an increase in the number of cases of the disease^6^.

Given this context, studies on the spatial distribution of vector-borne parasite infections which consider its relation to environmental risk factors are of great relevance for making decisions in epidemiological and environmental surveillance^7^.

Spatial data analyses in public health (ADES), based on kernel and Moran’s techniques, estimate the spatial dependence between epidemiological and environmental variables associated with malaria, contributing information on its incidence and conditions, and enabling the creation of several local scenarios for Brazil^8^.

Thus, this study aimed to analyze the socio-environmental production of malaria in the municipalities of Marabá, Parauapebas and Canaã dos Carajás, in the state of Pará, from 2014 to 2018.

## METHODS

This ecological, cross-sectional study on malaria was conducted in Marabá (233,669 inhabitants), Parauapebas (153,908) and Canaã dos Carajás (26,716), from 2014 to 2018. These municipalities are located in the Carajás Integration Region, in the state of Pará, and have significant demographic, environmental and socioeconomic gradients.

Epidemiological data (gender, age group, ethnicity, occupation, schooling, diagnosis, *Plasmodium* species, and parasitaemia) were obtained in the *Sistema de Informação de Agravo de Notificação* (Information System of Notifiable Diseases – SINAN) from the *Secretaria de Saúde do Estado do Pará* (State of Pará Health Department – SESPA). Environmental data on land use and cover were obtained from the TerraClass Program of the National Institute for Space Research (INPE). Cartographic data (municipal limits, protected areas, and indigenous lands) were obtained from the Census (2010) done by the Brazilian Institute of Geography and Statistics (IBGE).

Malaria cases and areas with pastures, secondary vegetation, forests, urbanization, agriculture and mining were georeferenced during fieldwork with the help of a global positioning system (GPS) receiver. After collecting data on the variables above, the information was treated to remove their inconsistencies and gaps via the TabWin 36b software, for subsequent storage in a geographic database (BDGEO).

Descriptive data analyses were performed by applying the nonparametric, statistical chi-square test of equal expected proportions, with significance set at 0.05%, via the Bioestat 5.0 software. During spatial data analysis (ADES), the distribution of the disease was analyzed via the kernel technique to identify the areas with the highest concentrations of cases in the municipalities. Spatial autocorrelation (I), between areas with malaria cases and different types of land use, was considered direct for I > 0, inverse for I < 0, and strong when indices were close to one of the defined variation limits (-1;1). The analysis was performed using the Moran’s global bivariate technique. The spatial analyses results were expressed by two thematic maps, produced via the Arcgis 10.5.1 software.

This study obtained approval no. 3,950,486/2019 from the Research Ethics Committee of the *Universidade do Estado do Pará* (State University of Pará), in accordance with the norms of Resolution no. 466/12 of the National Health Council.

## RESULTS

We analyzed 437 confirmed cases of malaria distributed thus: Marabá (154 cases), Parauapebas (248 cases), and Canaã dos Carajás (35 cases). The municipality of Parauapebas reported the greatest number of cases between 2014 and 2018, staying above the expected quantitative median for the whole period.

**Figure 1:**
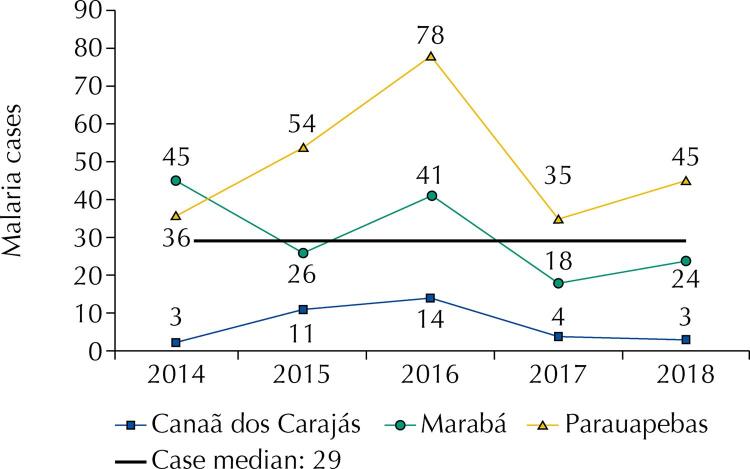
Historical series of malaria cases in the municipalities of Canaã dos Carajás, Marabá and Parauapebas, from 2014 to 2018.

Source: Research protocol, 2020.

The number of cases in Marabá fluctuated significantly during the observed period, despite remaining close to the median. The municipality of Canaã dos Carajás reported the least number of cases, staying below the average for the period. Figure 1 shows cases rose in 2016; year in which Parauapebas reported an outbreak.

Profile analysis of confirmed cases of malaria in the municipality of Marabá showed higher incidence among males (n = 115/74.68%), aged 18 to 59 years (n = 117/75.98%), of mixed race (n = 120/77.92%), attending up to elementary school (n = 102/66.23%) who were miners (n = 46/29.87%).

In the municipality of Parauapebas, the highest incidence of the disease also occurred in males (n = 198/79.84%), aged 18 to 59 years (n = 201/81.06%), of mixed race (n = 180/72.58%), attending up to elementary school (n = 114/45.97%) who were miners (n = 67/45.71%).

In Canaã dos Carajás, although the epidemiological profile was more significant among male individuals (n = 21/60%), aged 18 to 59 years (n = 27/77.16%), of mixed race (n = 29/82.87%) and attending up to elementary school (n = 20/57.14%), most were farmers (n = 16/45.71%).

All three municipalities showed a similar pattern of clinical variables. Thus, most common in the municipality of Marabá were the thick drop/smear diagnostic test (n = 147/95.45%), the *Plasmodium* vivax infection (n = 152/98.80%), and parasitaemia with two crosses (n = 149/96.75%). Parauapebas showed similar results: most common were the thick drop/smear test (n = 247/99.60%), the *Plasmodium* vivax species (n = 248/100.00%) and parasitaemia with two crosses (n = 241/97.18%), as did Canaã dos Carajás: most common were the thick drop/smear test (n = 35/100.00%), the *Plasmodium* vivax infection (n = 34/97.15%), and parasitaemia with two crosses (n = 33/94.28%). The Table shows the statistical significance of all variables analyzed (p < 0.05).

**Table:** Epidemiological profile of malaria cases in Marabá, Parauapebas and Canaã dos Carajás, Pará, from 2014 to 2018.

Variáveis		Marabá			Parauapebas			Canaã dos Carajás		

		n	%	p	n	%	p	n	%	p
Gender	**Male**	**115**	**74.68**	< 0.0001	**198**	**79.84**	< 0.0001	**21**	**60.00**	< 0.0001
	Female	39	25.32		50	20.16		14	40.00	

Age group	Child (0–11 years)	10	6.49	< 0.0001	28	11.29	< 0.0001	1	2.85	< 0.0001
	Teenager (12–18 years)	15	9.74		8	3.22		4	11.42	
	**Adult (19–59 years)**	**117**	**75.98**		**201**	**81.06**		**27**	**77.16**	
	Elderly (≥ 60 years)	12	7.79		11	4.43		3	8.57	

			0							

Race/Ethnicity	Indigenous	3	1.90	< 0.0001	0	0	< 0.0001	0	0	< 0.0001
	Asian	2	1.29		1	0.40		0	0	
	White	16	10.9		42	16.94		4	11.42	
	**Brown/Mixed race**	**120**	**77.92**		**180**	**72.58**		**29**	**82.87**	
	Black	13	8.01		25	10.08		2	5.71	

Occupation	Cattle production	28	18.18	< 0.0001	54	21.77	< 0.0001	**16**	**45.71**	< 0.0001
	Hunting/Fishing	2	1.29		7	2.82		0	0	
	Mining	**46**	**29.87**		**67**	**27.04**		5	14.28	
	Road/dam construction	3	1.94		20	8.06		1	2.88	
	Tourism	12	7.79		5	2.01		0	0	
	Domestic service	11	7.14		6	2.41		2	5.71	
	Other	**52**	**33.79**		**89**	**35.89**		11	31.42	

Education	Illiterate	2	1.29	< 0.0001	10	4.03	< 0.0001	3	8.57	< 0.0001
	**Elementary School**	**102**	**66.23**		**114**	**45.97**		**20**	**57.14**	
	High School	37	24.05		85	34.28		10	28.57	
	Higher Education	9	5.84		31	12.5		2	5.72	
	N/A	4	2.59		8	3.22		0	0	

Diagnosis	**Thick Drop/Smear**	**147**	**95.45**	< 0.0001	**247**	**99.6**	< 0.0001	**35**	**100**	< 0.0001
	Quick test	7	4.54		1	0.40		0	0	

*Plasmodium* species	***P. vivax***	**152**	**98.8**	< 0.0001	**248**	**100**	< 0.0001	**34**	**97.15**	< 0.0001
	*P. falciparum*	2	1.20		0	0		0	0	
	Mixed	0	0		0	0		1	2.85	

Parasitaemia	“+/2”	2	1.29	< 0.0001	1	0.40	< 0.0001	0	0	< 0.0001
	“+”	1	0.67		3	1.22		1	2.87	
	**“++”**	**149**	**96.75**		**241**	**97.18**		**33**	**94.28**	
	“+++”	0	0		1	0.40		0	0	
	“++++”	2	1.29		2	0.80		0	0	
	Unknown	0	0		0	0		1	2.85	

Analysis of the spatial distribution of malaria cases, based on the kernel technique, showed a heterogeneous pattern. While the municipalities of Marabá and Parauapebas showed a high density of cases in urban and periurban areas, density in Canaã dos Carajás remained average throughout its territory. Table 2 shows the very high percentage of cases reported in Parauapebas (56.8%), medium in Marabá (35.2%) and low in Canaã dos Carajás (8%).

Research also showed a very high anthropization percentage, related mainly to the presence of pastures, secondary vegetation, annual agriculture, and mining in the municipalities of Marabá (87.12%), Canaã dos Carajás (62.30%); but medium in Parauapebas (32.16%). Thus, we estimated, via Moran’s global bivariate technique, a possible direct and strong spatial autocorrelation between areas of disease notification, and municipality anthropization. As a result, Marabá and Parauapebas showed both a high number of cases and anthropization, while Canaã dos Carajás showed low values for both variables, as shown in [Fig f02] Figure 3.

**Figure 2 f02:**
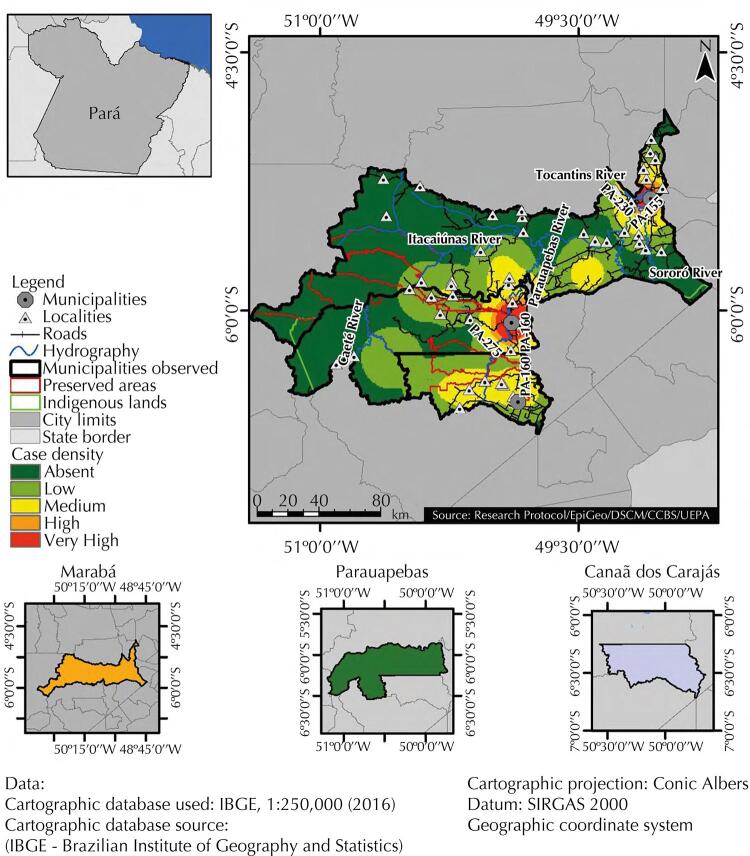
Density of malaria cases in the municipalities of Marabá, Parauapebas and Canaã dos Carajás, in Pará, from 2014 to 2018.

Source: Research protocol, 2020.

**Figure 3:**
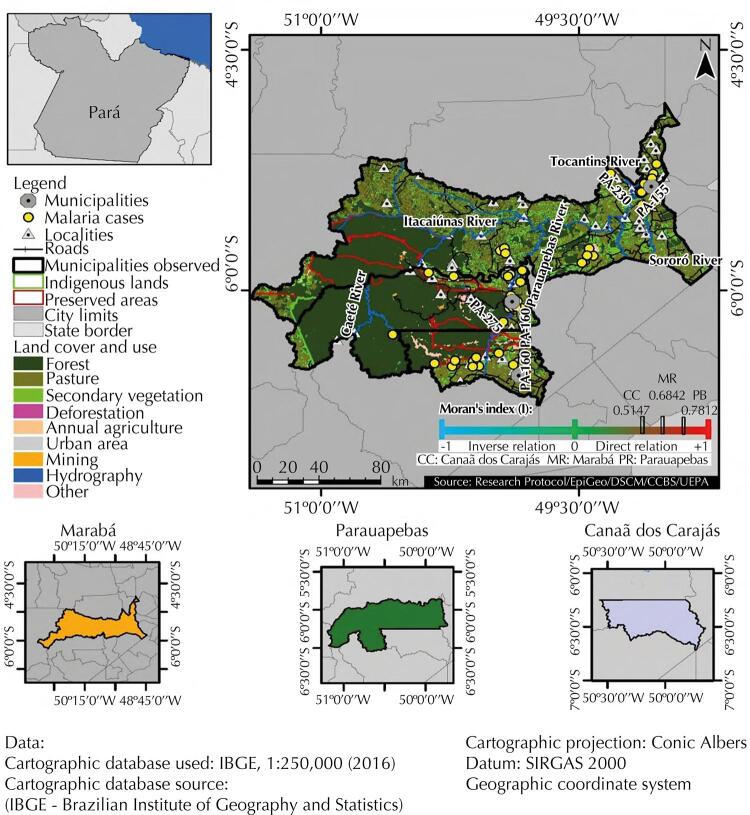
Spatial distribution of malaria cases and land use/cover in the municipalities of Marabá, Parauapebas and Canaã dos Carajas, Pará, from 2014 to 2018.

Source: Research protocol, 2020.

Xikrin indigenous lands had malaria cases, which - though located in protected areas of preserved rainforest - suffer great anthropic pressure, especially on its borders. We also identified the occurrence of cases in localities near preserved areas (*Tapirapé* Biological Reserve, *Tapirapé-Aquiri* National Forest, *Itacaiúnas* National Forest, *Apa do Igarapé Gelado, Carajás* National Forest and *Campos Ferruginososos* National Park), as well as high levels of anthropization at the limits of these areas (Figure 3).

## DISCUSSION

The municipalities of Marabá and Parauapebas showed a high number of confirmed cases of malaria for the period, following a pattern of recurrent endemicity in some portions of the state of Pará, especially those belonging to the Carajás Integration Region. This may be due to the intense migratory flow in the region, especially since 2014, given the implementation of industrial centers in Parauapebas and Marabá.

Our fieldwork shows that males, aged 19 to 59 years, working in the three municipalities observed, are possibly more affected by the disease given the absence of individual and collective protection, increasing their exposure to risk factors.

This suggests that, for the population in those areas, malaria is a work-related disease since exposure to risk factors is determined by the nature of their occupations. For example, most notifications in the municipalities of Marabá and Parauapebas show a close relationship between mining expansion and malaria transmission, since those workers are more exposed to malaria vectors, common in several areas of the Amazon^9^.

Our research showed that cattle production may also be related to malaria transmission cycles. In the municipality of Canaã dos Carajás, most individuals raised cattle, suggesting that the changes in the natural environment resulting from this activity are also risk factors for the disease, since pastures entail deforestation^10^.

According to the 2010 Census, 73% of the population of the state of Pará declare themselves of mixed race^11^, which explains the higher notification incidence on this population. This phenotypic characteristic refers to individuals of varied ethnic ancestry and may be related to Brazil’s historical miscegenation.

Low schooling in the three municipalities suggests that the lack of formal education impairs the understanding of the risks of malaria transmission, neglecting the disease. This also implies the need to raise awareness of the right to access to measures of individual and collective protection of the disease, such as methods to combat vectors and prophylactic measures^12^. The epidemiological profile observed points to the social vulnerability of this population, which can be mitigated via the development of formal and health education aiming at reducing exposure to risk factors^13^.

We used the thick drop/smear test for most diagnoses, as it is the most used in endemic areas, due to its sensitivity, faster staining, processing of large sample numbers, low cost, detection low-value parasitaemias (between 40 and 60 parasites per 100 microscopic fields), practicality, and application ease. However, the test requires experience in identifying species since the morphology of the parasite changes during sample discoloration^14^.

The greater occurrence of *Plasmodium* vivax may be associated with rapid gametocyte development in reservoirs, and the lack of drugs to treat the hypnozoite phase: the latent forms of the parasite during the hepatic cycle.

The observation of two crosses in parasitaemia (2 to 20 parasites per field, and 501 to 10,000 per mm^3^) may indicate a late diagnosis, hindering the treatment of unfavorable prognoses, and impairing the environmental and epidemiological surveillance of the disease, especially in more anthropized areas, in which land use is unsustainable, implicating in the production of a socio-environmental cycle for the disease, allowing an early treatment^12^.

The pattern of heterogeneous spatial distribution identified in the three municipalities may be related to different processes of human occupation and their consequent anthropic relations, influenced by socioeconomic dynamics, especially in Marabá and Parauapebas. They are characterized by the intense exploitation of natural resources, especially in mining and cattle production^17^. Thus, the formation of case clusters in these municipalities due to the intense migratory flow to these areas, and the subsequent uncontrolled population growth - facilitated malaria transmission cycles.

The region has a history of anthropic activity, especially in recent decades, with road construction, illegal logging, mining, and cattle production. The latter significantly favors a higher density of anopheline mosquitoes^18^, while the former shows the strong spatial autocorrelation between anthropized areas and malaria incidence.

Demarcating and maintaining preserved areas and indigenous lands, especially in the municipalities of Parauapebas and Canaã dos Carajás, was very important to prevent the intense, continuous deforestation; and preserve the Amazon, implicating in the reduction of malaria cases and breaking the transmission cycles caused by deforestation^11^.

## CONCLUSIONS

This study analyzed the connection between the socio-environmental and epidemiological variables related to the occurrence of malaria in Marabá, Parauapebas and Canaã dos Carajás, from 2014 to 2018. Our results show that the disease is a major public health issue in these municipalities, mainly due to the environmental production associated with land use and occupation.

The epidemiological profile of affected individuals was male, adult, of mixed race who attended up to elementary schooling and were involved mainly in mining and cattle production.

We observed a multifactoriality of malaria given the heterogeneous distribution of the disease, in which case clusters formed, associated with different levels of environmental degradation.

The spatial data analysis techniques used sufficed for interpreting the epidemiological scenarios of the disease, and we managed to identify and characterize the environmental risk factors for the disease in the areas studied. In this sense, the generated analyses provide public health administrators with information aimed at the continuous, systematic and procedural surveillance of malaria.

We emphasize the need to intensify the environmental and epidemiological surveillance of the spatial distribution of malaria, so as to mitigate this disease in the population of the studied municipalities, especially in areas of high susceptibility and social vulnerability.
