# Supplementary material for: The socio-environmental production of malaria in three municipalities in the Carajás region, Pará, Brazil
Source: Rev Saude Publica. 2021 Oct 18;55:73. doi: 10.11606/s1518-8787.2021055003463 (PMC8522715; doi:10.11606/s1518-8787.2021055003463)
Supplement: Translation

# Translation: A produção socioambiental da malária em três municípios da região de Carajás, Pará, Brasil

**Authors:** Alba Lucia Ribeiro Raithy Pereira, Claudia do Socorro Carvalho Miranda, Juan Andrade Guedes, Rafael Aleixo Coelho de Oliveira, Pedro Silvestre da Silva Campos, Vera Regina Da Cunha Menezes Palácios, Camylle Maia Costa Faria, Tainara Carvalho G. M. Filgueiras, Roberto Carlos Figueiredo, Nelson Veiga Gonçalves

**Affiliations:** I Brasil Universidade do Estado do Pará. Centro de Ciências Biológicas e da Saúde. Departamento de Saúde Comunitária, Laboratório de Epidemiologia e Geoprocessamento da Amazônia. Belém, PA, Brasil; II Brasil Universidade Federal Rural da Amazônia. Instituto Ciberespacial. Belém, PA, Brasil

**Keywords:** Malária, epidemiologia, Usos do Solo, Fatores Antropológicos, Fatores de Risco, Análise Espacial

## Abstract

**OBJETIVO:**

Analisar a produção ambiental da malária nos municípios de Marabá, Parauapebas e Canaã dos Carajás, no Pará, entre 2014 e 2018.

**MÉTODOS:**

Estudo ecológico e transversal a partir de dados epidemiológicos do Sistema de Informações de Vigilância Epidemiológica da Malária, da Secretaria de Saúde do Estado do Pará. Foram utilizados também dados cartográficos do Instituto Brasileiro de Geografia e Estatística (IBGE) e ambientais do projeto TerraClass, do Instituto Nacional de Pesquisas Espaciais (INPE). As análises estatísticas utilizaram o cálculo de percentuais e o teste qui-quadrado e as espaciais as técnicas de Kernel e de Moran global bivariado (I).

**RESULTADOS:**

Foram analisados 437 casos confirmados de malária, na área e periodo de estudo. O maior percentual de casos ocorreu em indivíduos do sexo masculino, adultos, morador da zona rural, com atividades de garimpagem e agropecuária, sendo o *Plasmodium vivax* a espécie de maior frequência e o diagnóstico mais utilizado a gota espessa/esfregaço. A distribuição da malária não ocorreu de forma homogênea, com evidências de dependência espacial entre áreas com ocorrência de casos e diferentes tipos de uso da terra. Foram observadas também autocorrelações espaciais relacionadas à alta variabilidade dos tipos antropismos, ocorrida nos municípios.

**CONCLUSÃO:**

A produção ambiental da malária está associada principalmente à pastagem e à mineração, antropismos relacionados às formas de uso e ocupação da terra nos municípios estudados. As tecnologias de análises de dados espaciais em saúde foram satisfatórias para a construção do cenário epidemiológico da doença.

## INTRODUÇÃO

A malária é uma doença infecciosa, febril aguda, de etiologia parasitária, cuja ocorrência envolve o homem, o protozoário do gênero *Plasmodium* e a fêmea infectada do mosquito do gênero *Anopheles* (Diptera: *Culicidae*). Esse agravo é considerado a principal doença parasitária do mundo, com expressivo número de casos nos países tropicais e subtropicais^1^.

Segundo a Organização Mundial da Saúde (OMS), a malária é endêmica em 105 países, com 228 milhões de casos notificados no ano de 2018 e causando 405 mil óbitos. Na América do Sul foram identificadas, nesse mesmo ano, regiões de expansão da doença na Colômbia, Equador, Venezuela e Brasil^4^.

Embora no Brasil tenha diminuído o número de casos a partir da década de 1960, a malária ainda apresenta um padrão de alta endemicidade, sendo considerada um complexo problema de saúde pública, devido às diferentes características ambientais, socioeconômicas e demográficas, sobretudo na Região Amazônica, onde foram notificados mais de 170 mil casos, apenas no ano de 2018^2^.

Somente no estado do Pará, localizado na parte oriental da Amazônia, notificou aproximadamente 115 mil casos de malária, entre 2014 e 2018, com tendência de aumento. O crescente número de casos, especialmente nos municípios de Canaã dos Carajás, Marabá e Parauapebas, localizados na Região de Integração do Carajás, tem relações estreitas com as alterações ambientais ocorridas nesse território, sobretudo mudanças relacionadas ao uso e cobertura da terra. O impacto da degradação ambiental sistemática e contínua torna esses três municípios emblemáticos quando se trata de produção ambiental da malária^5^_._

Dentre as principais causas dessas alterações ambientais estão as formas econômicas não sustentáveis e socialmente injustas de exploração dos recursos naturais, observadas de forma recorrente nestes três municípios, por meio de atividades como madeireira, agrária, pecuária bovina, minerária de ferro e de infraestrutura, que têm acarretado intensos fluxos migratórios, desmatamento extensivo e, consequentemente, aumento do número de casos da doença^6^.

Nesse contexto, os estudos relacionados à distribuição espacial de parasitoses transmitidas por vetores, como a malária, considerando a relação da doença com seus fatores de riscos ambientais, são de grande relevância para os processos de tomada de decisão em vigilância epidemiológica e ambiental^7^.

As Análises de Dados Espaciais em Saúde (ADES), baseadas na técnica de Kernel e de Moran, têm sido utilizadas para estimar a dependência espacial entre variáveis epidemiológicas e ambientais associadas à malária, contribuindo para a produção de informações sobre ocorrência e condicionantes, possibilitando a criação de diversos cenários em escalas locais no território brasileiro^8^.

A partir desse cenário, este trabalho procurou analisar a produção socioambiental da malária nos municípios de Marabá, Parauapebas e Canaã dos Carajás, no estado do Pará, entre 2014 e 2018.

## MÉTODOS

Este estudo ecológico e transversal sobre malária foi realizado em Marabá (233.669 hab.), Parauapebas (153.908 hab.) e Canaã dos Carajás (26.716 hab.), no período de 2014 a 2018. Esses municípios estão localizados na Região de Integração do Carajás, estado do Pará, e apresentam expressivos gradientes demográficos, ambientais e socioeconomicos.

Os dados epidemiológicos (sexo, faixa etária, etnia, ocupação, escolaridade, exame diagnóstico, espéce de *plasmodium* e parasitemia) foram adquiridos no Sistema de Informação de Agravo de Notificação (SINAN) da Secretária de Saúde do Estado do Pará (SESPA). Os dados ambientais (relacionado ao uso e cobertura da terra) foram obtidos no Programa TerraClass, do Instituto Nacional de Pesquisas Espaciais (INPE). Os dados cartográficos (limites municipais, unidades de conservação e de terras indígenas) foram obtidos no Censo Demográfico (2010), do Instituto Brasileiro de Geografia e Estatística (IBGE).

Os casos de malária e as áreas com Pastos, Vegetação Secundária, Florestas, Urbanização, Agricultura e Mineração foram georreferenciadas em campo com o auxílio de um receptor do Sistema de Posicionamento Global (GPS). Após o levantamento dos dados relacionados às variáveis acima, foi realizada a depuração das informações, com a retirada de inconsistências e incompletudes por meio do software TabWin 36b, para posterior armazenamento em um Banco de Dados Geográficos (BDGEO).

Foram realizadas análises descritivas dos dados, com a aplicação do teste estatístico não paramétrico qui-quadrado de proporções esperadas iguais, com significância de 0,05%, utilizando o programa Bioestat 5.0. Na Análise de Dados Espaciais (ADES), a distribuição da doença foi analisada com o uso da técnica de Kernel, para identificar as áreas com maiores concentrações de casos nos municípios. A autocorrelação espacial (I), entre as áreas com casos de malária e com diferentes tipos de uso da terra, foi considerada direta para I > 0 e inversa para I < 0 e forte quando os índices estavam próximos de um dos limites definidos de variação (-1 e 1), sendo essa análise realizada através da técnica de Moran global bivariado. Os resultados das análises espaciais foram expressos por dois mapas temáticos, produzidos com o software Arcgis 10.5.1.

Este estudo obteve o parecer favorável 3.950.486/2019, do Comitê de Ética em Pesquisa da Universidade do Estado do Pará, de acordo com as normas da Resolução nº 466/12 do Conselho Nacional de Saúde.

## RESULTADOS

Foram analisados 437 casos confirmados de malária distribuídos em Marabá (154 casos), Parauapebas (248 casos) e Canaã dos Carajás (35 casos). O município de Parauapebas notificou o maior número de casos entre 2014 e 2018, permanecendo acima do quantitativo médio de casos esperados para todo período.

Em Marabá houve uma variação expressiva no número de casos durante o período pesquisado, embora tenham ficado próximos da média. Já o município de Canaã dos Carajás apresentou o menor número de notificações, ficando abaixo da média de casos em todo o período. Observou-se que em 2016 houve aumento do número de notificações nos três municípios, mesmo ano em que Parauapebas notificou um surto de casos, conforme se verifica na Figura 1.

**Figura 1:** Série histórica dos casos de malária nos municípios de Canaã dos Carajás, Marabá e Parauapebas, entre 2014 e 2018.

Fonte: Protocolo de Pesquisa, 2020.

A análise do perfil dos indivíduos confirmados com malária no município de Marabá evidenciou maior percentual de ocorrência no sexo masculino (n = 115/74,68%), na faixa etária de 18 a 59 anos (n = 117/75,98%), cor parda (n = 120/77,92%), escolaridade até o ensino fundamental (n = 102/66,23%) e ocupação de garimpagem (n = 46/29,87%).

No município de Parauapebas os maiores percentuais da doença também ocorreram no sexo masculino (n = 198/79,84%), na faixa etária de 18 a 59 anos (n = 201/81,06%), cor parda (n = 180/72,58%), escolaridade até o ensino fundamental (n = 114/45,97%) e ocupação de garimpagem (n = 67/45,71%).

Já em Canaã dos Carajás, embora o perfil epidemiológico tenha sido mais expressivo em individuos do sexo masculino (n = 21/60%), na faixa etária de 18 a 59 anos (n = 27/77,16%), cor parda (n = 29/82,87%) e com escolaridade até o ensino fundamental (n = 20/57,14%), o maior percentual relacionado à ocupação dos doentes foi de agricultor (n = 16/45,71%).

Os três municípios apresentaram um padrão de similaridade em relação às variaveis clínicas. Assim, o tipo de exame diagnóstico mais utilizado no município de Marabá foi a gota espessa/esfregaço (n = 147/95,45%), com infecção por *Plasmodium* vivax (n = 152/98,80%) e parasitemia com duas cruzes (n = 149/96,75%). Resultado semelhante foi obtido em Parauapebas, onde os casos foram diagnosticados com exame gota espessa/esfregaço (n = 247/99,60%), espécie de *Plasmodium* vivax (n = 248/100,00%) e parasitemia com duas cruzes (n = 241/97,18%). A mesma tendência foi seguida no município de Canaã dos Carajás apresentando também de forma mais expressiva o diagnostico pela gota espessa/esfregaço (n = 35/100,00%), com infecção pelo *Plasmodium* vivax (n = 34/97,15%) e parasitemia com duas cruzes (n = 33/94,28%). Todas as variáveis analisadas nos três municípios de estudo apresentaram nivel de significancia estatistica (p–valor < 0,05), conforme mostra a Tabela.

**Tabela:** Perfil epidemiológico dos casos de malária em Marabá, Parauapebas e Canaã dos Carajás, Pará, no período de 2014 a 2018.

Variáveis		Marabá			Parauapebas			Canaã dos Carajás		

		n	%	p	n	%	p	n	%	p
Sexo	**Masculino**	**115**	**74,68**	< 0,0001	**198**	**79,84**	< 0,0001	**21**	**60,00**	< 0,0001
	Feminino	39	25,32		50	20,16		14	40,00	

Faixa etária	Criança (0–11 anos)	10	6,49	< 0,0001	28	11,29	< 0,0001	1	2,85	< 0,0001
	Adolescente (12–18 anos)	15	9,74		8	3,22		4	11,42	
	**Adulto (19–59 anos)**	**117**	**75,98**		**201**	**81,06**		**27**	**77,16**	
	Idoso (≥ 60 anos)	12	7,79		11	4,43		3	8,57	

			0							

Raça/Etnia	Indígena	3	1,90	< 0,0001	0	0	< 0,0001	0	0	< 0,0001
	Amarela	2	1,29		1	0,40		0	0	
	Branca	16	10,9		42	16,94		4	11,42	
	**Parda**	**120**	**77,92**		**180**	**72,58**		**29**	**82,87**	
	Preta	13	8,01		25	10,08		2	5,71	

Ocupação	Agropecuária	28	18,18	< 0,0001	54	21,77	< 0,0001	**16**	**45,71**	< 0,0001
	Caça/Pesca	2	1,29		7	2,82		0	0	
	Garimpagem	**46**	**29,87**		**67**	**27,04**		5	14,28	
	Construção de estradas/barragens	3	1,94		20	8,06		1	2,88	
	Turismo	12	7,79		5	2,01		0	0	
	Doméstica	11	7,14		6	2,41		2	5,71	
	Outros	**52**	**33,79**		**89**	**35,89**		11	31,42	

Escolaridade	Analfabeto	2	1,29	< 0,0001	10	4,03	< 0,0001	3	8,57	< 0,0001
	**Ensino Fundamental**	**102**	**66,23**		**114**	**45,97**		**20**	**57,14**	
	Ensino Médio	37	24,05		85	34,28		10	28,57	
	Educação Superior	9	5,84		31	12,5		2	5,72	
	Não se aplica	4	2,59		8	3,22		0	0	

Exame diagnóstico	**Gota espessa/Esfregaço**	**147**	**95,45**	< 0,0001	**247**	**99,6**	< 0,0001	**35**	**100**	< 0,0001
	Teste rápido	7	4,54		1	0,40		0	0	

Espécie de *Plasmodium*	***P. vivax***	**152**	**98,8**	< 0,0001	**248**	**100**	< 0,0001	**34**	**97,15**	< 0,0001
	*P. falciparum*	2	1,20		0	0		0	0	
	Mista	0	0		0	0		1	2,85	

Parasitemia	“+/2”	2	1,29	< 0,0001	1	0,40	< 0,0001	0	0	< 0,0001
	“+”	1	0,67		3	1,22		1	2,87	
	**“++”**	**149**	**96,75**		**241**	**97,18**		**33**	**94,28**	
	“+++”	0	0		1	0,40		0	0	
	“++++”	2	1,29		2	0,80		0	0	
	Ignorado	0	0		0	0		1	2,85	

A análise da distribuição espacial dos casos de malária, baseada na técnica de Kernel, apresentou um padrão não homogêneo de ocorrência, enquanto nos municípios de Marabá e Parauapebas houve densidade muito alta de casos concentrada nas áreas urbanas e periurbanas, em Canaã dos Carajás a densidade manteve-se média em toda a extensão territorial. A maior ocorrência de casos foi notificada em percentuais muito altos em Parauapebas (56,8%), médios em Marabá (35,2%) e baixos em Canaã dos Carajás (8%). Como demonstra a Figura 2.

**Figura 2:** Densidade de casos de malária nos municípios de Marabá, Parauapebas e Canaã dos Carajas, Pará, entre 2014 e 2018.

Fonte: Protocolo de Pesquisa, 2020.

A pesquisa revelou ainda percentuais muito altos de antropização, principalmente relacionados à presença de pastos, vegetação secudária, agricultura anual e mineração, nos municípios de Marabá (87,12%), Canaã dos Carajás (62,30%) e médio em Parauapebas (32,16%). Assim, estimou-se uma possível autocorrelação espacial direta e forte entre as áreas de notificação da doença e as antropizações nos municípios, utilizando a técnica de Moran global bivariado. Como resultado, Marabá e Parauapebas apresentaram alto número de casos e alta antropização, enquanto que Canaã dos Carajás apresentou baixa ocorrência nas duas variáveis, conforme apresenta a Figura 3.

**Figura 3:** Distribuição espacial de casos de malária e o uso/cobertura da terra nos municípios de Marabá, Parauapebas e Canaã dos Carajas, Pará, entre 2014 e 2018.

Fonte: Protocolo de pesquisa, 2020.

Verificou-se ainda a ocorrência de malária em terras indígenas Xikrim, que ficam em áreas protegidas de floresta ombrófila preservada, ainda que haja grande pressão antrópica, principalmente à margem desse território. Foi identificado também a ocorrência de casos em localidades próximas às unidades de conservação ambiental (Reserva Biológica do Tapirapé, Floresta Nacional do Tapirapé-Aquiri, Floresta Nacional do Itacaiúnas, Apa do Igarapé Gelado, Floresta Nacional do Carajás e Parque Nacional dos Campos Ferruginosos), além de níveis altos de antropização nos limites dessas áreas (Figura 3).

## DISCUSSÃO

Os municípios de Marabá e Parauapebas apresentaram números elevados de casos confirmados de malária no período estudado; acompanhando um padrão de endemicidade recorrente em alguns territórios do estado do Pará, sobretudo naqueles pertencentes à região de integração do Carajás, isso pode estar relacionado ao intenso fluxo migratório ocorrido para esta região, de forma mais expressiva a partir de 2014, devido à implantação dos polos industriais em Parauapebas e Marabá.

O maior acometimento de indivíduos do sexo masculino em idade produtiva (19 a 59 anos), nos três municípios de estudo, possivelmente se relaciona ao fato de os homens executarem maior número de atividades laborais sem o uso de proteção individual e coletiva, o que aumenta o grau de exposição aos fatores de risco da doença, como observou-se nos trabalhos de campo.

Esse fato sugere que nas áreas estudadas podemos considerar a malária uma doença relacionada ao trabalho, devido aos diferentes níveis de exposição ou contato direto das populações humanas com os fatores de riscos, determinados pela natureza de suas ocupações. Por exemplo, a garimpagem foi a atividade ocupacional mais expressiva dentre as notificações nos municípios de Marabá e Parauapebas, o que demonstra uma relação estreita entre a expansão da garimpagem com os ciclos de transmissão da malária, já que nessa atividade laboral as pessoas estão mais expostas ao contato direto com os vetores da malária, que são muito comuns em diversas áreas da Amanzônia^9^.

Esta pesquisa revelou que a atividade agropecuária também pode estar relacionada a ciclos de transmissão da malária, no município de Canaã dos Carajás a maioria dos indivíduos estavam inseridos em atividades agropecuárias, sobretudo a criação de gado, sugerindo que as alterações no ambiente natural decorrentes dessas atividades também se constituem fatores de risco da doença, cujo principal condicionante é o desmatamento para criação de pastos, como se observou em determinadas áreas do município^10^.

Segundo o Censo 2010, 73% da população do estado do Pará se autodeclara parda^11^, explicando a maior incidência de notificações da malária sobre essa parcela da população. Essa característica fenotípica refere-se aos indivíduos com variadas ascendências étnicas e ancestrais e pode estar relacionada à miscigenação histórica ocorrida entre mulatos, caboclos e cafuzos.

A baixa escolaridade evidenciada nos três municípios sugere que a falta de educação formal dificulta o entendimento dos riscos de transmissão da malária, negligenciando a doença, isso implica também na necessidade da conscientização do direito ao acesso às medidas de proteção individual e coletiva da doença, como métodos de combate aos vetores e medidas profiláticas^12^. O perfil epidemiológico observado aponta para a vulnerabilidade social dessas populações, que pode ser mitigada a partir do desenvolvimento de educação formal e em saúde, objetivando a diminuição da exposição aos fatores de risco^13^.

A maior parte dos diagnósticos encontrados neste estudo foram feitos com a técnica da gota espessa/esfregaço, pois é o exame parasitológico direto mais utilizado nas áreas endêmicas da doença, devido sua sensibilidade, coloração mais rápida, processamento de grande número de amostras, baixo custo, possibilidade de detecção de parasitemias mesmo com valores baixos (entre 40 e 60 parasitos por 100 campos), praticidade e facilidade na realização. Contudo, esse exame requer experiência para a identificação de espécies, uma vez que a morfologia do parasito se altera durante o processo de desemoglobinização^14-16^.

O maior acometimento de casos pela espécie *Plasmodium vivax* pode estar associado ao rápido desenvolvimento dos gametócitos (forma infectante para o mosquito) nos reservatórios e à falta de medicamentos para tratar a fase de hipnozoítos, que são formas latentes do parasito no ciclo hepático.

A observação de duas cruzes na parasitemia (2 a 20 parasitos por campo e 501 a 10.000 por mm^3^) pode indicar diagnóstico tardio da doença, que dificultam o tratamento dos casos com prognóstico desfavorável, o que aumenta o desafio para a vigilância ambiental e epidemiológica com busca ativa de casos, principalmente nas áreas com maior nível antropização, decorrente do uso não sustentável da terra, implicando a produção de ciclo socioambiental da doença, sendo, assim, possível o tratamento da malária ainda em fase inicial^12^.

O padrão de distribuição espacial não homogêneo identificado nos municípios de estudo pode estar relacionado à ocorrência de diferentes processos de ocupação humana e suas consequentes relações antrópicas, influenciadas pela dinâmica socioeconômica, principalmente de Marabá e Parauapebas. Essas atividades são caracterizadas por intensa exploração dos recursos naturais, principalmente nos processos de extração de minérios e a produção agropecuária^17^. Assim, a formação de aglomerados de casos nas sedes destes municípios foi influenciada pelo intenso fluxo migratório de pessoas para essas áreas, ocasionando ocupação desordenada, facilitando o estabelecendo de ciclos de transmissão da malária.

A região a que pertencem os municípios estudados é historicamente afetada por atividades antrópicas, principalmente nas últimas décadas, com contruções de estradas, exploração ilegal de madeira, mineração, além da agropecuária, que favorece expressivamente o aumento da densidade de anophelinos^18^, e da garimpagem, o que explixa a autocorrelação espacial direta e forte entre as áreas com antropismos e a incidência da malária.

A demarcação e manutenção das unidades de coservação e das terras indígenas, principalmente nos municípios de Parauapebas e Canaã dos Carajás, foi muito importante para impedir o intenso processo contínuo de desflorestamento e se mostram efetivas na preservação do ambiente natural amazônico, implicando na diminuição de casos de malária e quebrando os ciclos de transmissão causados pelo desmatamento^11^.

## CONCLUSÕES

Neste estudo, analisou-se a conexão entre as variáveis socioambientais e epidemiológicas relacionadas à ocorrência da malária em Marabá, Parauapebas e Canaã dos Carajás, entre 2014 e 2018. Os resultados evidenciaram que a doença é um grande problema de saúde pública nesses municípios, principalmente em decorrência da produção ambiental associada ao uso e ocupação da terra.

O perfil epidemiológico dos indivíduos acometidos foi sexo masculino, adultos, pardos, escolaridade até o ensino fundamental e cujas principais atividades ocupacionais são a garimpagem e a agropecuária.

Foi observada uma multifatorialidade da malária ao notar-se uma distribuição não homogênea da doença, com a formação de aglomerado de casos associados a níveis diferenciados de degradação ambiental.

As técnicas de análises de dados espaciais utilizadas foram satisfatórias para a interpretação dos cenários epidemiológicos da malária, sendo possível identificar e caracterizar os fatores de riscos ambientais para a doença, nas áreas de estudo. Nesse sentido, as análises geradas permitem prover os gestores em saúde de informações voltadas para a vigilância contínua, sistemática e processual do agravo.

Ressaltamos a necessidade de intensificar as ações de vigilância ambiental e epidemiológica, para monitorar a distribuição espacial da malária, como forma de mitigação dessa doença nas populações humanas dos municípios de estudo, principalmente as que estão nas áreas de alta suscetibilidade e apresentam vulnerabilidade social.
